# Supplementary material for: A meta-analysis of circulating microRNAs in the diagnosis of papillary thyroid carcinoma
Source: PLoS One. 2021 May 21;16(5):e0251676. doi: 10.1371/journal.pone.0251676 (PMC8139519; doi:10.1371/journal.pone.0251676)
Supplement: S1 Table — (PDF) [file pone.0251676.s001.pdf]

S1 Table. The basic characteristics of Inclusion literatures

Serial number	First author	Publication time	Research country	Target microRNA	source of miRNAs	method	Internal reference	Tp	Fp	Fn	Tn
1	Yu(a)[17]	2012	Asia	Let-7e/miR-222 /miR-151-5p	serum	qRT-PCR	miR-16	93	11	13	84
2	Yu(b)	2012	Asia	Let-7e	serum	qRT-PCR	miR-16	67	10	39	85
3	Yu(c)	2012	Asia	miR-151-5p	serum	qRT-PCR	miR-16	63	10	43	85
4	Yu(d)	2012	Asia	miR-222	serum	qRT-PCR	miR-16	86	10	20	85
5	Cantara(a)	2014	Europe	miR-95	serum	qRT-PCR	miR-16	75	1	4	79
6	Cantara(b)	2014	Europe	miR-29b	serum	qRT-PCR	miR-16	38	12	41	68
7	Cantara(c)	2014	Europe	miR-190	serum	qRT-PCR	miR-16	74	17	5	63
8	Cantara(d)	2014	Europe	miR-579	serum	qRT-PCR	miR-16	62	12	17	68
9	Lee(a)[19]	2015	Asia	miR-146b	plasma	qRT-PCR	Cel-miR-39	43	8	27	11
10	Lee(b)	2015	Asia	miR-155	plasma	qRT-PCR	Cel-miR-39	52	7	18	12
11	Li(a)[20]	2015	Asia	miR-25-3p	plasma	qRT-PCR	U6 RNA	52	30	4	65
12	Li(b)	2015	Asia	miR-451a	plasma	qRT-PCR	U6 RNA	50	32	6	63
13	Li(c)	2015	Asia	miR-25-3p/miR-451a	plasma	qRT-PCR	U6 RNA	54	34	2	61
14	Yu(a)[21]	2016	Asia	miR-124-3p	plasma	qRT-PCR	miR-16	44	12	6	38
15	Yu(b)	2016	Asia	miR-9-3p	plasma	qRT-PCR	miR-16	35	18	15	32
16	Yu(c)	2016	Asia	miR-196b-5p	plasma	qRT-PCR	miR-16	37	17	13	33
17	Rosignolo(a)	2017	Europe	miR-146a-5p	serum	qRT-PCR	Cel-miR-39	32	9	8	10
18	Rosignolo(b)	2017	Europe	miR-221-3p	serum	qRT-PCR	Cel-miR-39	16	0	24	19
19	Rosignolo(c)	2017	Europe	miR-222-3p	serum	qRT-PCR	Cel-miR-39	19	3	21	16
20	Zhang(a)[23]	2017	Asia	miR-222/miR-221/miR-146b	serum	RT-qPCR	miR-16	85	1	21	34
21	Zhang(b)	2017	Asia	miR-222	serum	RT-qPCR	miR-16	67	4	39	31
22	Zhang(c)	2017	Asia	miR-221	serum	RT-qPCR	miR-16	91	16	15	19
23	Zhang(d)	2017	Asia	miR-146b	serum	RT-qPCR	miR-16	100	11	6	24
24	zhang[24]	2017	Asia	miR-451	serum	qRT-PCR	Cel-miR-39	28	10	42	60
25	zhang(a)[25]	2018	Asia	miR-222	serum	qRT-PCR	miR-16	35	3	23	32
26	zhang(b)	2018	Asia	miR-221	serum	qRT-PCR	miR-16	45	17	13	18
27	zhang(c)	2018	Asia	niR-146b	serum	qRT-PCR	miR-16	45	7	13	28
28	zhang(d)	2018	Asia	MiR-21	serum	qRT-PCR	miR-16	51	4	7	31
29	zhang(e)	2018	Asia	miR-222/miR-221/miR-146b/miR-21	serum	qRT-PCR	miR-16	50	2	8	33

Abbreviation: Tp, true positives; Fp, false positives; Fn, false negatives; Tn, ture negatives.
