# Supplementary material for: A meta-analysis of circulating microRNAs in the diagnosis of papillary thyroid carcinoma
Source: PLoS One. 2021 May 21;16(5):e0251676. doi: 10.1371/journal.pone.0251676 (PMC8139519; doi:10.1371/journal.pone.0251676)
Supplement: S2 Table — (PDF) [file pone.0251676.s002.pdf]

S2 Table. Subgroup analysis of circulating microRNAs in the diagnosis of PTC

Subgroup	study numbers	Sen (95%CI)	Spe (95%CI)	PLR (95%CI)	NLR (95%CI)	DOR (95%CI)	AUC (95%CI)
**Area**							
Asia	22	0.80 (0.73 - 0.85)	0.79 (0.73 - 0.85)	3.9 (2.9 - 5.1)	0.26 (0.20-0.34)	15 (10 - 23)	0.86 (0.83 - 0.89)
Europe	7	0.75 (0.54 - 0.88)	0.88 (0.74 - 0.95)	6.4 (2.6-15.8)	0.28 (0.14-0.58)	22 (6 - 88)	0.90 (0.87 - 0.92)
**The number of miRNAs**							
1	25	0.76 (0.69 - 0.82)	0.80 (0.74 - 0.85)	3.9 (2.9-5.1)	0.29 (0.22-0.39)	13 (8 - 21)	0.85 (0.82 - 0.88)
>1	4	0.88 (0.80 - 0.94)	0.89 (0.73 - 0.96)	8.1 (3.2-20.7)	0.13 (0.08-0.21)	62 (29- 136)	0.94 (0.92 - 0.96)
**The type of miRNA**							
miR146b	3	0.82 (0.60 - 0.93)	0.71 (0.60 - 0.79)	2.8 (1.8-4.2)	0.26 (0.10-0.66)	11 (3 - 38)	0.72 (0.68 - 0.76)
miR-222	3	0.69 (0.57 - 0.79)	0.90 (0.84 - 0.94)	6.8 (4.1-11.4)	0.34 (0.24-0.49)	20 (10 - 42)	0.90 (0.84 - 0.94)
**source of miRNA**							
serum	21	0.76 (0.68 - 0.83)	0.86 (0.80 - 0.91)	5.6 (3.8-8.0)	0.27 (0.20-0.38)	20 (12 - 35)	0.89 (0.86 - 0.91)
plasma	8	0.83 (0.73 - 0.90)	0.66 (0.61 - 0.71)	2.5 (2.0-3.0)	0.25 (0.15-0.43)	10 (5 - 19)	0.68 (0.64 - 0.72)
**Internal reference**							
miR-16	20	0.80 (0.74 - 0.85)	0.84 (0.78 - 0.89)	5.1 (3.6-7.3)	0.24 (0.18-0.32)	22 (13 - 38)	0.89 (0.86 - 0.92)
others	9	0.74 (0.56 - 0.86)	0.75 (0.67 - 0.81)	2.9 (2.4-3.5)	0.35 (0.21-0.59)	8 (5 - 15)	0.79 (0.75 - 0.82)

Subgroup analysis was performed by area, number of microRNA, types of microRNA, sample source of microRNA and expression trend of microRNA compared with control group, $p<0.05$ was considered significant.

Abbreviation: DOR: diagnostic odds ratio; AUC: The area under the curve.

6
