# Supplementary figures and images for: A meta-analysis of circulating microRNAs in the diagnosis of papillary thyroid carcinoma
Source: PLoS One. 2021 May 21;16(5):e0251676. doi: 10.1371/journal.pone.0251676 (PMC8139519; doi:10.1371/journal.pone.0251676)

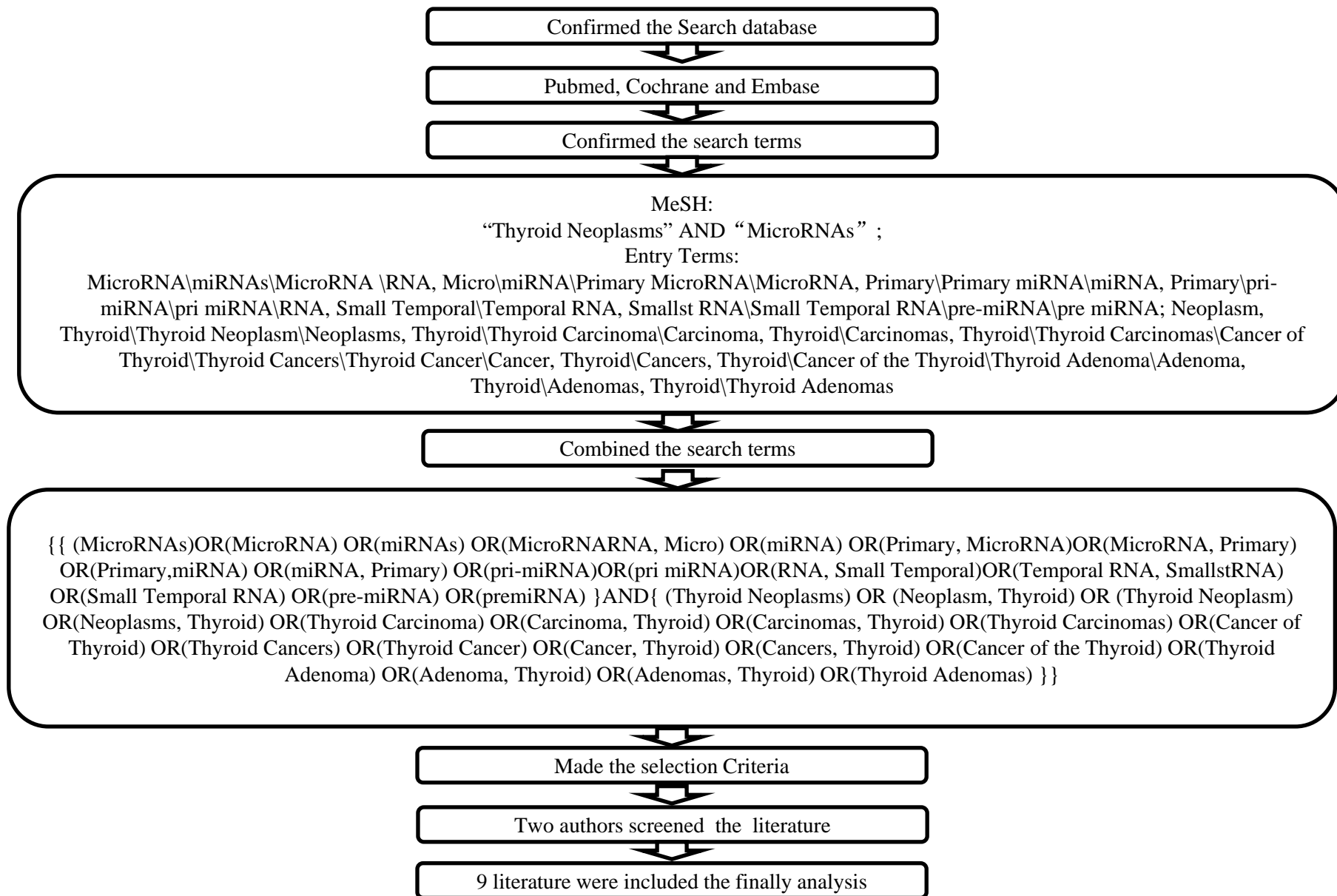

Supplement: S2 File — (PDF) [file pone.0251676.s004.pdf]
